# Posttraumatic stress disorder and its associated factors among people living in Dabat district, northwest Ethiopia

**DOI:** 10.3389/fpsyt.2024.1359382

**Published:** 2024-07-30

**Authors:** Mihret Melese, Lemlemu Maru, Dereje Esubalew

**Affiliations:** ^1^ Department of Human Physiology, School of Medicine, College of Medicine and Health Science, University of Gondar, Gondar, Ethiopia; ^2^ Department of Human Physiology, College of Medicine and Health Sciences, Ambo University, Ambo, Oromia Region, Ethiopia

**Keywords:** stress related disorders, trauma, war, Ethiopia, prevalence

## Abstract

**Background:**

The conflict between the Ethiopian government and the Tigray People’s Liberation Front (TPLF) in the Dabat district of Ethiopia has led to significant civilian casualties, instances of rape, sexual abuse, and property theft. These traumatic events contribute to the development of post-traumatic stress disorder (PTSD) among local residents. However, there is currently no available data on the prevalence of PTSD and its associated factors in this region. This study seeks to fill this gap by assessing PTSD prevalence and identifying related factors among residents of the war-affected Dabat district in northwest Ethiopia.

**Method:**

A community-based correctional study was conducted in the Woken and China kebeles of Dabat district, northwest Ethiopia, spanning from July 13 to September 19, 2023. A total of 410 participants were selected using systematic random sampling, making a 100% response rate. The study utilized an interviewer-administered questionnaire, which included the Post-Traumatic Stress Disorder Checklist (PCL-5) to assess PTSD. The research investigated the association between PTSD and various demographic and psychosocial characteristics using both bivariate and multivariable binary logistic regression analyses. Statistical significance was set at a P-value of 0.05.

**Results:**

The majority of participants in the study were male (62%) with a mean age of 33 ( ± 1.67) years. The overall prevalence of PTSD was 30.7% (95% CI: 26.6–35.10). Multivariable logistic regression analysis identified several factors significantly associated with PTSD: symptoms of depression (AOR=3.5; 95% CI: 1.13-6.89), age between 45 and 67 years (AOR=1.68; 95% CI: 1.04-5.78), experiencing stressful life events (AOR=1.63; 95% CI: 1.05-7.86), experiencing sexual abuse or rape (AOR=1.53; 95% CI: 1.07-6.75), chewing khat (AOR=1.48; 95% CI: 1.08-4.56), being female (AOR=1.43; 95% CI: 1.13-3.67), and having an income of 34.6 USD (AOR=1.28; 95% CI: 1.07-4.67).

**Conclusion and recommendation:**

This study reported that the prevalence of PTSD was high. As a result, the study suggested that governments and other stakeholders should be involved in implementing efficient interventions and quick measures to mitigate the effects of war on mental health following the conflict. The government and nongovernmental organizations were also advised by these studies to continue providing humanitarian assistance, which should include access to food, clean water, clothing, shelter, and education. This study also suggested that people living in conflict zones should be legally protected from rape, sexual abuse, arson, detention without cause, and kidnapping.

## Introduction

The Tigray conflict in Ethiopia lasted from November 2020 to November 2022 and was mainly concentrated in the Tigray region (1). It involved the Ethiopian federal government and the Tigray People Liberation Front. The Ethiopian government declared war following accusations of Tigray forces attacking its northern command base (2). The conflict, which initially started in Tigray, later extended to neighbouring regions like Afar and Amhara, impacting over 20 million people, predominantly women and children. Approximately 5.5 million individuals were displaced, fleeing to other parts of Ethiopia (3). The situation, particularly in Dabat District in northwest Ethiopia, worsened displacement, exposing many to homelessness and various criminal acts, including murder, sexual abuse, rape, and abduction. This significantly increased the risk of posttraumatic stress disorder among those affected.

Posttraumatic stress disorder is a highly prevalent mental disorder in war-affected areas or natural disasters (4–6). Posttraumatic stress disorder can result from experiencing or witnessing distressing events such as murder, threats, kidnapping, loss of loved ones, displacement from one’s home, and deprivation of basic needs such as food (7). It manifests symptoms such as nightmares and flashbacks, avoiding triggers connected to trauma, elevated alertness, and detrimental cognitive alterations (8). Post-war settings are frequently characterized by instability, peril, and a scarcity of humanitarian aid, factors that may contribute to the onset of posttraumatic symptoms (9, 10). In the absence of effective management, Posttraumatic stress disorder can lead to a deterioration in quality of life, disruptions in daily functioning, and, in severe cases, even mortality (11).

Posttraumatic stress disorder is more prevalent in low- and middle-income countries where mental health services are often less accessible (12). Worldwide, the documented prevalence of Posttraumatic stress disorder among global populations affected by war is 12.9% (13). There is significant regional variation in Posttraumatic stress disorder prevalence, ranging from 0.3% to 8.7% (14, 15). A systematic review across 40 nations found that 30.6% of internally displaced individuals affected by war experienced Posttraumatic stress disorder (16). War-affected regions of sub-Saharan Africa also show high rates, with reported prevalence reaching up to 30% (17). In Uganda, Posttraumatic stress disorder prevalence is identified at 11.8% In Uganda (18), whereas in Kenya, it notably rises to 62.1% (18), South Sudan has reported a prevalence of 28% (19). Within Ethiopia, there is significant variation in Posttraumatic stress disorder rates: Addis Ababa reports a lower rate of 3.7% (20), while Dessie reports a higher prevalence of 34.5% (5).

Several factors contribute to the development of posttraumatic stress disorder following exposure to wartime trauma. These factors include age, gender, experiences of potentially traumatic events during and after the war, unemployment, lower educational levels, and the presence of chronic diseases (19, 21–23).

Limited information exists regarding the prevalence of mental disorders, particularly posttraumatic stress disorder, among individuals directly involved in armed conflicts in low- and middle-income countries such as Ethiopia. However, a substantial number of individuals in such contexts are vulnerable to various mental health challenges due to their exposure to conflict-related trauma (24, 25). In the Dabat district of Ethiopia, a substantial conflict involving the Ethiopian government and the TPLF erupted, leading to numerous civilian casualties, incidents of rape and sexual abuse, cessation of hostilities, and property theft. These traumatic events may have contributed to the development of posttraumatic stress disorder among the affected population. However, available data on the prevalence of posttraumatic stress disorder and its associated risk factors among individuals residing in conflict-affected areas post-war are lacking. Therefore, this study aimed to investigate the extent of posttraumatic stress disorder and identify factors associated with it. The findings from this research have the potential to inform the government and other stakeholders, guiding them in implementing interventions and appropriate actions to address the lingering issues following the war.

## Methods

### Study area and period

This study was conducted in Woken and China Kebeles, which are located in the Dabat district of the Amhara regional state in Northwest Ethiopia, spanning from July 13 to September 19, 2023. The Dabat district is located approximately 775 kilometres away from Addis Ababa. These two kebeles comprise 2000 households with a total population of 8,000. Unfortunately, during the study period, China and Woken Kebeles became battlegrounds between the Tigray People’s Liberation Front and Ethiopian government forces. The Amhara Media Corporation documented that China and Woken Kebeles witnessed the tragic loss of hundreds of civilians, either through massacres or shelling. Furthermore, many residents endured severe hardships, including instances of sexual abuse and rape.

### Study design and population

A community-based cross-sectional study was carried out within war-affected regions of the Dabat district, specifically in China and Woken Kebeles. The source population consisted of adult residents aged 18 and above living in China and Woken Kebeles, who had been residing in the two kebeles for at least six months. However, the target population or study population included all adult individuals aged 18 years and above who presented themselves during the data collection period.

### Sample size calculation

By applying a single proportion population formula, the sample size was determined using the estimated prevalence rate of PTSD, which was 58.4%, as identified in a study conducted in southern Ethiopia (26).


$$ni=\frac{{\left(\text{Zα}/2\right)}^{2}*p\left(1-p\right)}{d^{2}}=\frac{{\left(1.96\right)}^{2}*0.584\;\left(1-0.584\right)}{{\left(0.05\right)}^{2}}=374$$

With the 10% nonresponse rate added, the total sample size was 412 with a 5% margin of error, a 95% confidence level, and a 10% nonresponse rate.

### Sampling technique and procedure

**Figure 1:**
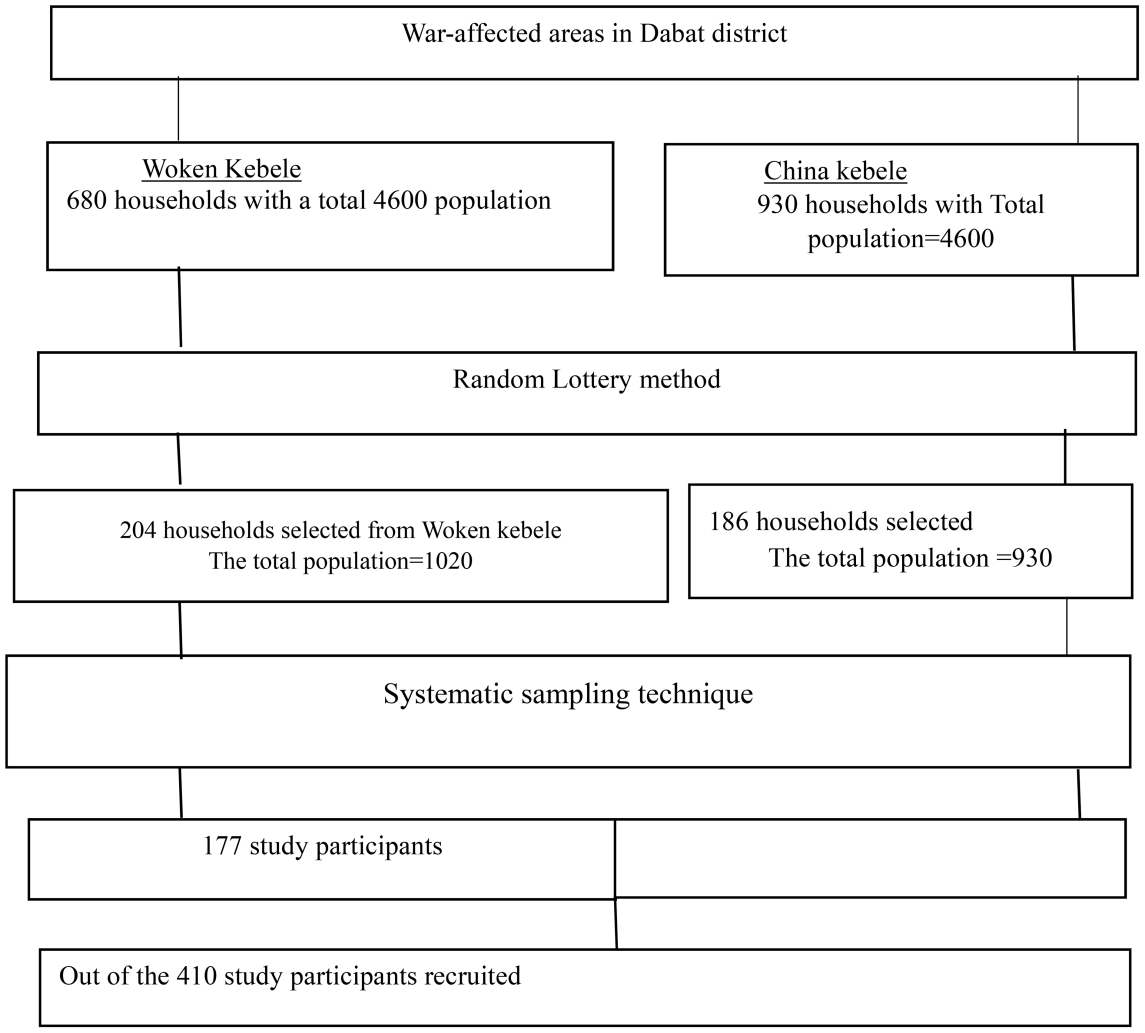
sampling procedure for the war-affected area in the Dabat district, northwest Ethiopia, 2023.

Participants for the study in both China and Woken Kebeles were recruited using a systematic random sampling technique. Initially, the number of households and the total population were determined for both kebeles. Then, 30% of households were selected from each kebele. Proportional allocation was applied to the selected households. Finally, the sampled population was chosen through a systematic random sampling technique after proportional allocation (refer to **Figure 1**).

### Eligibility criteria and study variables

The inclusion criteria for this study included all adult individuals aged 18 years or older, residing in China and Woken Kebele for a minimum duration of 6 months.

Individuals who reported being severely ill and unable to participate in interviews for data collectors, those who provided a medical certificate or related document indicating their current illness, and those with observable verbal communication problems were excluded from the study.

Posttraumatic stress disorder: A 20- item post-traumatic checklist (PCL-5) was used to assess PTSD). A cumulative score was derived by summing the 20 items, with scores ranging from 0 to 80 on a five-point Likert scale (0 = not at all, 1 = a little bit, 2 = moderately, 3 = quite a bit, and 4 = extremely), Which means adults who score< 50 have no PTSD and ≥ 50 have PTSD (27).

#### Depression

Participants exhibiting depression were identified through their scores on the Patient Health Questionnaire (PHQ), specifically those individuals who scored 10 or more on the nine items (28).

Stressful life event. It was determined by the presence of one or more life-threatening events within the last six months, as indicated by the relevant questions (29).

#### Social support

Social support was assessed using the Social Support Scale (OSS-3), which consists of three items with scores ranging from 3 to 14. A score within the range of 3–8 indicates poor social support, while a score between 9–11 suggests intermediate social support. A score of 12–14 on the OSS-3 reflects strong social support (30).

Substance abuse was defined as the consumption of cigarettes, alcohol, or khat by individuals who met the criteria of (31, 32).

### Data collection tools

Data collection was conducted by psychiatric professionals with Bachelor of Science (BSc) degrees, trained in data collection techniques and ethical principles. The questionnaire was initially developed in English and translated into Amharic, with a back-translation into English to ensure accuracy. Training was provided to study participants about the study’s objectives, handling ambiguous questions, and adhering to ethical standards, including informed consent, confidentiality, anonymity, and data management. PTSD was assessed using the 20-item self-report Post-Traumatic Stress Disorder Checklist (PCL-5), which measures the 20 DSM-5 PTSD symptoms. We validated this assessment tool internally, achieving a Cronbach’s alpha reliability coefficient of 0.78. It has been previously utilized in Ethiopia (5, 20). The Validity and reliability of the PCL-5 had been tested and proven in a different country, for example, Zimbabwe (Cronbach’s alpha = 0.92) with sensitivity and specificity of 74.5% and 70.6% respectively (33), (5). We validated this assessment tool, achieving a Cronbach’s alpha reliability coefficient of 0.78. Depression was measured using the Patient Health Questionnaire-9 (PHQ-9), where a person is considered depressed if their score is 10 or higher on the nine items. The PHQ-9 has 4 items with corresponding scores: not at all (0), several days (34), more than half a day (15), nearly every day (35), and over the last week (28).

### Data processing and statistical analysis

The collected data was coded and entered into Epi-Data version 4.6, followed by analysis using IBM SPSS Statistics version 25.0 software. Summary statistics, including proportions and frequencies, were utilized to summarize the results in tables. Bivariable and multivariable logistic regression models was fitted to identify factors associated with the outcome variable. In the bivariable logistic regression, variables with a p value ≤ 0.2 were considered candidates for inclusion in the multivariable logistic regression. Binary logistic regression was employed. Adjusted odds ratios (ORs) was used to determine the relationships between PTSD and associated variables at a significance level of p ≤ 0.05. The normality of continuous data was assessed using the Shapiro−Wilk test, and the model’s fitness was checked using the Hosmer–Lemshow goodness-of-fit test.

### Ethics approval and consent to participate

We obtained ethical approval from the Institutional Review Board of the University of Gondar, School of Medicine, College of Medicine, and Health Sciences Ethical Review Committee, with reference number IRB/373/2023, subsequent to the review of our research proposal. After adequately briefing the educated study participants about the study, written informed consent was obtained from each individual. Conversely, during data collection from illiterate study participants, the data collectors began by reading aloud the consent form, providing a thorough explanation of the study’s objectives and procedures. Upon participants’ voluntary agreement to participate, the data collectors marked “Yes” on the consent form. Subsequently, participants confirmed their consent by providing their signature using a “thumbprint” as acknowledgment. This approach was approved by the Institutional Review Board (IRB) of the University of Gondar. To uphold confidentiality and privacy, participants’ names and other personal identifiers were excluded from the documentation. To maintain confidentiality, potential identifiers were omitted from the questionnaire, and all collected data were securely stored. Our procedures adhered to the relevant rules and guidelines outlined in the Helsinki Declaration (36).

## Results

### Background characteristics of the study participants

In this study, a total of 410 study participants were included, making 100% response rate. The majority of participants were male (62%). The mean age of the study participants was 33 years ( ± 1.67). Approximately 45% of the participants were married, and 43% had completed elementary school. Nearly 63% of participants resided in rural areas, and approximately 45% were employed as farmers. Majority the participants reported a monthly income ranging from 34.6 USD (see **Table 1**).

**Table 1:** background characteristics of study participants who reside in the ware affected area in the Dabat district, northwest Ethiopia, 2023.

Study variables	Category	Frequency	Percentage
Gender	Male	254	62
	Female	156	38
Age (years)	18-25	139	33.9
	26-35	95	23.2
	36-45	93	22.7
	45-67	83	20.2
Marital status	Single	166	40.5
	Married	178	43.4
	Divorced or windowed	66	16.1
Educational status	Illiterate	58	14.1
	Primary schooling (Garde1-8)	174	42.4
	Secondary schooling (Grade 9-12)	104	25.4
	Diploma and above	74	18
Occupational status	Waged employed	59	14.4
	Farmer	183	44.6
	Unemployed	94	22.9
	Self employed	74	18.1
Income	>2000 ETB(34.6 USD)	253	61.7
	2001-3500ETB(34.6- 60.6 USD)	61	14.9
	>3500 ETB(>60.7 USD)	97	23.4
Place of residence	Urban	134	32.7
	Rural	276	63.3

### Clinical and behavioural characteristics of the study participants

In the present study, 17% of all participants reported a family history of mental illness. Approximately one-third of the participants (32%) had a known diagnosis of a chronic medical condition, 43% of whom were specifically diagnosed with diabetes. Symptoms of depression and morbidity were exhibited by approximately thirty percent of the research participants. More than thirty percent of the participants had engaged in khat chewing at some point in their lifetime. Additionally, one-third of the study participants reported experiencing sexual abuse or rape during the war (refer to **Table 2**).

**Table 2:** Clinical and behavioural factors among study participants who reside in the ware-affected area in the Dabat district, northwest Ethiopia, in 2023.

Variables	Category	Frequency	%
Clinical factors			
Known family history of diagnosed with mental illness	Yes	71	17.3
	No	339	82.6
Known diagnosed chronic medical condition	Yes	131	32
	No	279	68
Type of medical condition	Diabetic Mellitus	56	43.1
	Hypertension	41	31.6
	Cancer	18	13.8
	Heart disease	15	11.5
Depressive Symptoms	Yes	126	30.7
	No	284	69.3
Morbidity (incidence of health issues or conditions associated with PTSD).	Yes	123	30
	No	287	70
Behavioural factors			
Ever khat used in lifetime	Yes	121	29.5
	No	289	70.5
Current khat used in the last 3 months	Yes	96	23.4
	No	314	76.6
Ever used alcohol drinks in life	Yes	219	53.4
	No	191	46.6
Current used alcohol drinks in last 3 months	Yes	201	49
	No	209	51
Current tobacco products used in the last 3 months	Yes	67	16.3
	No	343	83.7
Sexually abused or raped	Yes	142	34.6
	No	268	65.4
Had ill health without medical care	Yes	166	40.5
	No	244	59.5
Experienced forced separation from family	Yes	185	45.1
	No	225	54.9
Experienced trauma/raped in childhood	Yes	176	42.9
	No	234	57.1
Stressful Life Event	Yes	62	15.1
	No	348	84.9
Social Support	Poor social support	135	32.9
	Intermediate social support	165	40.2
	Strong social support	110	26.8

### Prevalence of posttraumatic stress disorder

In this study, 30.7% of the participants (126 out of 410) had posttraumatic stress disorder (95% CI 26.6–35.1).

### Factors associated with posttraumatic stress disorder

Bivariable and multivariable logistic regression analyses of PTSD were conducted, as detailed in **Table 3**. In the bivariable analysis, sex, age, marital status, monthly income, educational status, khat chewing status, alcohol consumption status, family history of mental illness, depression symptoms, sexual abuse or rape status, and stressful life events were considered candidate variables for the multivariable analysis (p ≤ 0.2). Multivariable logistic regression analysis revealed several statistically significant factors associated with PTSD: having symptoms of depression (AOR=3.5; 95% CI: 1.13-6.89), age 45-67 years (AOR=1.68; 95% CI: 1.04-5.78), experiencing stressful life events (AOR=1.63; 95% CI: 1.05-7.86), experiencing sexual abuse or rape (AOR=1.53; 95% CI: 1.07-6.75), chewing khat (AOR=1.48; 95% CI: 1.08-4.56), being female (AOR=1.43; 95% CI: 1.13-3.67),an d having an income 34.6 USD (AOR=1.28; 95% C: (1.07-4.67). Symptoms of depression were the strongest predictor (AOR=3.5; 95% CI: 1.13-6.89), indicating that individuals with depression symptoms were 3.5 times more likely to develop PTSD compared to those without. Age 45-67 years (AOR=1.68; 95% CI: 1.04-5.78), showing that participants in this age group were 1.68 times more likely to develop PTSD compared to those aged 18 to 25 years. Stressful life events (AOR=1.63; 95% CI: 1.05-7.86), indicating a 1.63 times higher likelihood of PTSD among those who experienced such events. Sexual abuse or rape (AOR=1.56; 95% CI: 1.07-6.75), showing a 1.56 times greater odds of PTSD among individuals who experienced these incidents. Khat chewing (AOR=1.47; 95% CI: 1.08-4.56), indicating a 1.47 times higher likelihood of PTSD among khat chewers compared to non-chewers. Female gender (AOR=1.43; 95% CI: 1.13-3.67), with females having a 1.43 times greater likelihood of PTSD compared to males. Compared to study participants with incomes with of >60.7 USD, those with less than 34.6 USD had a higher likelihood of developing PTSD (AOR=1.28; 95% CI: 1.07-4.67).

**Table 3:** factors associated with PTSD among study participants who reside in the ware-affected area in the Dabat district, northwest Ethiopia, 2023.

Variables	Category	PTSD		COR (95%CI)	AOR (95%CI)	P value
		Yes	No			
		N (%)	N (%)			
Gender	Female	37 (23.7)	119 (76.3)	1.75 (1.10-2.71)	1.43 (1.13-3.67) *	0.03
	Male	89 (35)	165 (65)	1	1	1
Age (years)	18-25	25 (18)	114 (82)	1	1	1
	26-35	38 (45.8)	45 (54.2)	0.55 (0.29-1.02)	0.23 (0.13-0.78)	0.67
	36-45	36 (60.2	57 (39.8	0.04 (0.24-0.098)	0.57 (0.72-0.89)	0.59
	46-67	27 (28.4)	68 (71.6)	3.18 (1.25-8.08)	1.68 (1.04-5.78) *	0.02
Marital status	Single	74 (44.6)	92 (55.4)	1	1	
	Married	30 (16.9)	148 (83.1)	3.96 (2.41-6.52)	1.45 (0.12-5.63)	
	Widowed or divorced	22 (33.8)	43 (66.2)	1.57 (1.09-2.89)	1.17 (0.76-3.87)	
Monthly income (ETB)	<2000 ETB (34.6 USD)	33 (26.4)	92 (73.6)	2.86 (1.14-6.84)	1.28 (1.07-4.67) *	0.045
	2001-3500ETB (34.7-60.6 USD)	66 (36.8)	113 (63.2)	1.957 (1.15-3.32)	1.2 (0.16-5.45)*	0.45
	>3500 ETB (>60.7 USD)	27 (25.5)	79 (74.5)	1.32 (1.60-3.67)	1	1
Educational status	Illiterate	17 (29.3)	41 (70.7)	12.56 (10.54-22.55)	3.4 (0.67-6.98)	0.53
	Primary schooling	47 (27)	127 (73)	7.89 (5.45-12.34)	0.98 (0.45-578)	0.32
	Secondary schooling	35 (33.7)	69 (66.3)	2.56 (1.89-5.68)	1.22 (0.34-4.67)	0.065
	Diploma and above	27 (36.5)	47 (73.5)	1	1	1
Khat chewing	Chewer	33 (42.9)	44 (57.1)	1.94 (1.16-3.24)	1.47 (1.08-4.56) *	0.02
	No-chewer	93 (31.1)	239 (68.9)	1	1	1
Alcohol drinking	Drunker	82 (37.4)	137 (62.6)	2.00 (1.29-3.08)	3.3 (0.16-4.76)	0.12
	Non drunker	44 (23)	147 (77)	1	1	1
Family history of mental illness	Yes	44 (34.9)	88 (65.1)	5.08 (2.96-8.72)	1.3 (0.78-7.86)	0.71
	No	27 (9.5)	256 (90.5)	1	1	1
Depression symptoms	Yes	68 (54)	58 (46)	4.56 (2.90-7.19)	3.5 (1.13-6.89) **	0.001
	No	58 (20.4)	226 (79.6)	1	1	1
Sexual abused or raped	Yes	32 (25.4)	94 (74.6)	1.85 (1.16-2.96)	1.56 (1.07-6.75) *	0.001
	No	110 (38.7)	174 (61.3)	1	1	
Stressful life event	Yes	12 (9.5)	114 (90.5)	**2.**03 (1.04-3.96)	1.63 (1.05-7.86) *	0.1001
	No	50 (17.6)	234 (82.4)	1	1	1

1= reference category, Hosmer–Lemshow = 0.24, *p ≤ 0.05, ^**^p ≤ 0.001.

## Discussion

The observed prevalence of PTSD among people living in war-affected areas in Dabat district, was determined to be 30.7% (95% CI 26.6–35.1). These finding suggest a higher prevalence of PTSD among residents of war-affected regions in the study settings, this result is similar to findings from studies conducted in Dessie, Ethiopia (34.5%) (5), Israel (27%) (37), Servia (32.3%) (38) and South Sudan (28%) (19). However, this result is lower than those of studies conducted in Kenya, which reported a prevalence of 62.1% (39); in South Sudan Juba, which reported a prevalence of 37.6% (40); and in Croatia, which reported a prevalence of 56.7% (41). On the other hand, this prevalence is higher than that reported in studies conducted in Uganda, 18% (21), and in Libya 25.23% (42). In the Dabat district of Ethiopia, cultural beliefs profoundly influence responses to war-related atrocities such as rape, torture, murder, and abduction. Stigma often prevents victims from seeking justice or support services due to fears of social ostracization and reputation damage, potentially exacerbating PTSD development. Societal pressures and familial honor heavily influence whether victims disclose their experiences and the type of community support they receive. Additionally, religious and spiritual beliefs shape interpretations of trauma, affecting coping mechanisms and attitudes toward seeking professional help versus traditional healing practices (26, 43–45).

This study revealed that PTSD was positively associated with having symptoms of depression, ages 45-67, experiencing stressful life events, enduring sexual abuse or rape, chewing khat, being female, and having an income of less than 34.60 USD.

Symptoms of depression were the strongest predictor, showing that individuals with depression symptoms were more likely to develop PTSD compared to those without. This study is supported by a study performed in Nigeria (46), northeaster Ethiopia (5), the Maikadra massacre in Ethiopia (47),. This is because people with depression may have heightened sensitivity to stress and trauma, impaired coping mechanisms, and neurobiological changes that increase their vulnerability to developing PTSD (48). Elderly study participants were more prone to developing PTSD than their male counterparts were. This result is in line with research conducted in Netherland (49). This could be attributed to the greater prevalence of chronic health conditions such as heart disease, hypertension, and diabetes among elderly people, coupled with challenges such as sleep disturbances, insomnia, physical and mental fatigue, changes in appetite, muscle tension, pain, chest discomfort, an upset stomach, and other issues more commonly experienced in the elderly population. All these factors may contribute to the development of posttraumatic stress disorder (50, 51).

According to the results of this study, women were more likely to develop PTSD than men were. These findings align with research conducted in northeast Ethiopia (5) and Uganda (52).This could be attributed to the greater vulnerability of females to sexual abuse or rape during times of war, making them more susceptible to developing PTSD than men are (53).

A potential explanation could be the influence of sex hormones and neuro-steroids on emotional learning and memory formation, coupled with disparities in brain anatomy and activation in response to traumatic stress (54). Contrary to current findings, research conducted in the US found no significant difference in PTSD rates between men and women, even after adjusting for demographic factors and lifetime trauma exposure (55). Hence, it is reasonable to deduce that when males and females experience similar types and levels of traumatic stressors, disparities in PTSD rates between the genders may not be as pronounced (53). Individuals with lower incomes were more likely to develop PTSD than their higher-income counterparts were. This study aligns with the study conducted in low-income countries (56). This can be attributed to the vulnerability of individuals with low incomes, who are more prone to experiencing depression, anxiety, and increased levels of the hormone cortisol. These factors collectively contribute to heightened stress levels (57). Compared with no chewers, study participants who chewed khat had greater odds of developing PTSD. This study is supported by a study performed in Somali (58). A possible explanation is that chewing khat can result in side effects such as headaches, vertigo, decreased cognitive function, fine tremors, sleeplessness, heightened alertness, dependence, tolerance, and anxiety. All these effects could contribute to the development of posttraumatic stress disorder (59).

Compared to study participants who were not sexually abused or raped, those who suffered these events had a greater chance of developing PTSD. This study is in line with previous findings in the Balkans (4). This correlation may be attributed to the impact of depression, which influences susceptibility to trauma and contributes to the development of posttraumatic stress disorder by elevating steroid hormones such as cortisol (60). Finally, participants who experienced stressful life events exhibited a greater likelihood of developing posttraumatic stress disorder (PTSD) than did those who had not experienced such events. This study consistent with findings from a prior investigation conducted in southern Sudan (40) and northeast Ethiopia (5). This can be explained by the fact that stressful life events, such as problems with work, relationships, or finances, can exacerbate PTSD (61).

### Strengths and limitations of the study

The strength of this study was its use of primary or original data, which increased its relevance. This study and this study had certain limitations, including social desirability bias, as participants might be hesitant to provide socially acceptable responses to sensitive questions concerning sexual abuse and substance use. Additionally, there was a recall bias due to the limitations of the study design.

### Conclusion and recommendation

The results of this study showed that a significant proportion of people living in war-affected areas had PTSD. As a result, the study suggested that governments and other stakeholders should be involved in implementing efficient interventions and quick measures to mitigate the effects of war on mental health following the conflict. The government and nongovernmental organizations were also advised by these studies to continue providing humanitarian assistance, which should include access to food, clean water, clothing, shelter, and education. This study also suggested that people living in conflict zones should be legally protected from rape, sexual abuse, arson, detention without cause, and kidnapping. This study recommends that scholars employ a combination of qualitative and quantitative approaches to gain a comprehensive understanding of PTSD in war-affected areas. Furthermore, it highlights the critical role of cohort studies in establishing causal relationships between exposure to war and the development of PTSD.

## Data availability statement

The datasets presented in this study can be found in online repositories. The names of the repository/repositories and accession number(s) can be found in the article/Supplementary Material.

## Ethics statement

The studies involving humans were approved by the institutional review board of the University of Gondar’s School of Medicine, College of Medicine, and Health Sciences ethical review committee, with the reference number (IRB/373/2023). Upon explaining the study’s significance to participants, both verbal and written informed consent were obtained. To maintain confidentiality, potential identifiers were omitted in the questionnaire, and the collected data was securely stored. All procedures adhered to the relevant rules and guidelines of the Helsinki Declaration. The studies were conducted in accordance with the local legislation and institutional requirements. The participants provided their written informed consent to participate in this study.

## Author contributions

MM: Writing – original draft, Writing – review & editing. LM: Data curation, Conceptualization, Writing – original draft, Writing – review & editing. DE: Conceptualization, Writing – original draft, Writing – review & editing.
